# Academic, Activist, or Advocate? Angry, Entangled, and Emerging: A Critical Reflection on Autism Knowledge Production

**DOI:** 10.3389/fpsyg.2021.727542

**Published:** 2021-09-28

**Authors:** Monique Botha

**Affiliations:** Division of Psychology, University of Stirling, Stirling, United Kingdom

**Keywords:** autism, critical reflection, ableism, epistemic injustice, research violence, social justice, participatory research, dehumanization

## Abstract

There has been a focus on autistic-led and participatory research in autism research, but minimal discussion about whether the field is hospitable to autistic involvement. While the focus on participatory and/or autistic-led research is abundantly welcome, a wider conversation should also happen about how autistic people are treated in the process of knowledge creation. As such, I present a critical reflection on my experiences of academia as an autistic autism researcher. I open by questioning whether I am an academic, an activist, or an advocate before discussing my journey through academia, and my exposure to dehumanizing, objectifying, and violent accounts of autism. I highlight how the construction of objectivity has resulted in a failure to question the validity of these dehumanizing accounts of autism, which are regarded as “scientifically-sound” by virtue of their perceived “objectivity.” Furthermore, I discuss how the idea of objectivity is used to side-line autistic expertise in disingenuous ways, especially when this knowledge challenges the status-quo. Despite claiming to be value-free, these dehumanizing accounts of autism embody social and cultural values, with a complete lack of transparency or acknowledgment. I then discuss how these dehumanizing accounts and theories—entangled in values—reverberate into autistic people's lives and come to be ways of constituting us. Following this, I discuss the rationality of the anger autistic people feel when encountering these accounts, and instead of urging people to distance themselves from these emotions, I discuss the value of “leaning-in” as a radical act of dissent in the face of research-based violence. I then make a call to action urging all those who write or speak about autism to engage reflexively with how their values shape their understanding and construction of autistic people. Lastly, I conclude by answering my opening question: I have emerged as an advocate, activist, and academic. For me, belonging to the autistic community, acknowledging our marginalization, and recognizing our suffering within society means that hope for a better and just future has always, and will always underpin my work.

## Introduction

Let me introduce myself—I am an autism academic. I am first generation university educated, saddled with student debt, and carry the uncomfortable feeling that I do not fully belong most of the time. I did my MSc, followed by my Ph.D. at the University of Surrey, and before this I did my BA in Social Care Practice at Athlone Institute of Technology in Ireland. I worked for 4 years with autistic children and young people, and their families, as a social care practitioner, as well as doing palliative care for young and middle-aged disabled adults. I am unashamedly, and unabashedly autistic. I have been the kind of autistic that was “going nowhere,” “disruptive,” “awkward,” and “failing,” and I have been the kind of autistic that is “inspirational,” “going places,” and “changing the world.” I have been the kind of autistic that melted down every day, until I was pinned down on the ground being sedated in the middle of my hometown. Some have described me as being a “dead-end,” and the “kind of person with autism who was born to die by suicide anyway,” and also as the kind of person with the “easy autism.” I am very honest, but mask most of the time and walk a delicate line between “personal” and “professional.” Somewhere along the way I decided to be an autism academic, but first I was just autistic, then an advocate, then I was an activist, all before the academy told me to leave those at the door.

In this article, I want to open the door to discussing knowledge production, and what it means to do research into autism. As involvement of autistic people is hopefully increasing in research, blunt and open conversation is needed to address how autistic involvement is received, and whether the field is hospitable for us. As such, I publish this in the hope that it contributes to a conversation on what is needed to ensure equal engagement in research from autistic people in the field of autism research. Furthermore, it has been my experience that autistic scholars at all stages experience a loneliness that silence serves—we are not meant to speak openly about our experiences within academia, especially when negative. It is my hope that on publishing this autoethnographic account, some autistic scholars might feel less alone.

My MSc and Ph.D. research were into the utility of the minority stress model for understanding poor mental health in the autistic community (Botha and Frost, 2020), and whether autistic community connectedness would buffer against the effect of minority stress on mental health (Botha, 2020). I used qualitative and quantitative methods, and did four studies—a qualitative, critical grounded theory investigation into autistic community connectedness; a scale creation and evaluation study for measuring autistic community connectedness; a cross-sectional investigation into whether community moderated the effect of minority stress on mental health in autistic people; and finally, a longitudinal study investigating the effect of minority stress and autistic community connectedness over time. I write this article—somewhere between personal and professional, open, and unambiguous, in the hopes I can spark a wider conversation on autism, objectivity, and positionality—a conversation that needs to happen amongst anyone who researches autism. While there has been wider discussions about participatory and autistic-led research (something I am deeply in favor of myself) (Botha, accepted,i; Pellicano, 2014; Fletcher-Watson et al., 2019), it seems no one has stopped to ask whether autism research is, at its *core* hospitable to autistic involvement, nor fully explored the damage often done to autistic people in their involvement.

## Positivism, Objectivity Psychology, and Autism

Elsewhere I have discussed in more depth how positivism has shaped psychology and in turn, the construction of autism (Botha, accepted) and so here, I will keep this section short. In essence, mainstream psychology has been underpinned by positivism and logical empiricism for most of its relatively short history—this means that in general, psychology aims for establishing reality through the application of the scientific methods (Leahey, 1992). These methods are designed to aim for falsification, deduction, and establishing causality (Popper, 2008). Positivism is predicated on “epistemological transcendence” (Nagel, 1989); the idea that employing the scientific method means that the end product is value-free mean that it is, untied to social and cultural values (Fondacaro and Weinberg, 2002). Objectivity then, is distance from the object. Yet—no one discusses *how* objectivity is *functionally* achieved—instead most quantitative research forgoes discussion of objectivity all together under the assumption that the work simply stands alone. Given this, some have described objectivity as a “useless elevator concept” that is ideal in theory but not does not work in practice (Hacking, 2015).

The history of autism is rooted in the field of medicine (and by extension psychiatry) which tends to treat deviation from the norm as disease, disorder, and dysfunction, and which tends to have a focus on remediation, prevention, and cure (Glynne-Owen, 2010; Evans, 2013). The enshrinement of the idea of the scientific method, positivism, and objectivity within fields like medicine has resulted in both a bio-essentialism and pathologisation of autism, whereby autism at its worst is described as an epidemic (Johansen, 2013). This “disease” or “disorder” is identified through a set of observable behaviors (according to the DSM-5 impairment in social communication, impairment in social interaction, and lastly restrictive and repetitive behaviors), all of which should have been noticeable from a young age. Furthermore, within this medical model, remediation, prevention, and curing should be the primary goals of research—akin to the treatment of other “disorders.” Therefore, early interventions, such as applied behavioral analysis, that strive to normalize the perceived deviances of autism are extolled as gold standard interventions.

Autism is not necessarily a natural category—it is a label that was created by technocrats to group together a cohort of people with similar behavioral presentations (Hacking, 2001; Silberman, 2015). As I have highlighted elsewhere (Botha, in review) autism cannot be explained as emerging from biology alone, despite the best efforts of positivism; genes are found in a wide array of combinations, and this is an evolving and ever-changing combination (De Rubeis et al., 2014), while at a neurobiological level autistic brains are highly heterogenous (Toal et al., 2010; Lenroot and Yeung, 2013; Chapman, 2020). Autism is so heterogenous that some argue that it is no longer meaningful as a single category (Happé et al., 2006; Mottron, 2021). This does not mean that autism is not real—on the contrary I believe, given the knowledge that we have, that 1 day a biological explanation which underpins autistic people will emerge (explaining the sensory differences that unite us, for example (see Proff et al., 2021, for a recent review). What this means instead, is that the actual *meaning* of autism has been something long-debated and shaped by people during its 100-year history, and as such, autism has always been tied to *time, place*, and *culture*. Thus, even if tomorrow, we were to uncover a specific array of genes, or a specific part of the brain that was reliable and valid across the entire all autistic people, we *still* would not understand autism if we did not consider society or culture.

## Navigating Autism as a Paradigm

### Undergraduate

As an undergraduate in my penultimate year, my *academic* introduction to autism was in a module entitled “Abnormal Psychology” through the triad of impairments (Wing and Potter, 2002) which categorized “people with autism” as being marked by impairments in our social communication and language, social interaction, and as having restricted interests and cognitive inflexibility. I was taught about autistic people having impaired theory of mind (Baron-Cohen et al., 1985), and told that “people with autism” would struggle to understand the perspective, experiences, and emotions of others—I was well-acquainted with the Sally-Ann task as evidence of my deficiency. I was introduced to the idea that impaired theory of mind meant that autistic people struggled with empathy (Baron-Cohen, 2009b). I was repeatedly told to use person-first language (“person with autism”) because identity-first language was out-dated, offensive, and reduced a “person with autism” to their autism alone—“we *must* remember this is a person first.” I was taught that autism was a linear scale from “severely autistic” to “mild autism” like Asperger syndrome or “high-functioning autism.” “There is no cure” was how I was introduced to Applied Behavioral Analysis as the only scientifically-sound treatment for autism—the goal of which was to teach children to bridge across their intrinsic deficits and into non-autistic communication and sensibility.

I would learn these two-dimensional, seemingly objective accounts of autistic people on one day, and on the next work with these three-dimensional autistic children who were all together more complicated, and more real. Autistic children who were sensory-seeking, inquisitive, and who creatively used social communication to get their needs met only to be chastised for not using *more* words. I would spend countless hours online on various forums with other autistic people building up friendships, asking for advice, giving advice, and quite literally *sharing perspectives* with others like me—and a lot of the stories were of trauma, marginalization, mental health crises, and about the way autistic people were treated in society. But as I tried to express my own experiences as an autistic in class I would be shut down because of my “lack of objectivity,” and because “I could not possibly put myself in the shoes of the person with *severe* autism.” I spent a lot of time being taught that I lacked theory of mind by people who could not grasp that my experience of and with autism were fundamentally different to the accounts being taught. I discovered Steven Kapp's paper on identity first language and neurodiversity (Kapp et al., 2013) and it brought me a deep-seated joy and certainty because it was so much closer to the reality I was witnessing. I tried to elevate it into discussions only to be told that “I did not understand the literature” and “was not qualified to challenge it.” So, when my exams came around, I rote-learned my own dehumanization and rattled off a list of deficits and early-intervention behavioral modifications tools to be used on people like me to pass the exam. I went home and melted down. I graduated with a first-class honor degree, being told that if I were *really* autistic, I probably would have dropped out along the way.

### Postgraduate

My MSc research was my first foray into creating academic autism knowledge. I did not intend to do my MSc research on autism—my undergraduate project was on the knowledge and perception that Irish citizens held toward asylum seekers in Ireland, and I thought I would continue down a similar vein. I wanted to do equality and value-based research but did not feel like I had a place amongst autism research. Two things happened which changed my course: firstly, a study was published showing that autistic people have increased early mortality and one of the leading causes of death is suicide (Hirvikoski et al., 2016)—a paper which shook me to my core. Secondly, at the University of Surrey, my paths crossed with an academic who would introduce me to the concept of minority stress (Meyer, 2003). The minority stress model posits that social disadvantage and marginalization results in an increased burden, which in turn can result in mental and physical health disparities (Meyer et al., 2002; Frost et al., 2015). Predominantly, it has been used to investigate the health disparities seen in the queer community. The focus in the minority stress model shifts away from there being something inherent about LGBTQ+ communities and focuses instead on the experiences that sexual and gender minorities have within society. It sounds cliché, but it was a light-bulb moment—it was a lens through which I could reflect on an entire lifetime of experiences and make them coherent for once. Yet, as an idea, minority stress ran counter to the literature which associated the traits of autism itself with suicidality (Mikami et al., 2009), centered suffering as inherent to autism (Baron-Cohen and Bolton, 1993), or focused on the specific thinking styles of autistic people as causative of poor mental health—as if autistic people exist in a societal blackhole, and would still suffer in the absence of our entire social structure.

It is not hard to see the potential utility for the minority stress model when you pause and take stock of how autistic people are treated in society. The minority stress model captures the some of the complexity of existing while autistic. Autistic people are stereotyped—and the vast majority of stereotypes are negative (Wood and Freeth, 2016). Autistic people face employment discrimination, higher unemployment, and underemployment, as well as experiencing bullying in the workplace (Shattuck et al., 2012; Baldwin et al., 2014). Autistic children are more likely to be excluded from schools (Timpson and Great Britain, 2019). In the United Kingdom (UK), one-third of autistic people have access to neither employment or welfare payments (Redman, 2009), while 12% of Welsh autistic adults report experiencing homelessness (Evans, 2011). Statistics show disproportionate use of force against autistic people and those with learning disability in the UK (Home Office, 2018), while a third to half of all incidents involving the use of excessive force by police involves a disabled person (Perry and Carter-Long, 2016)—experiences which will obviously be further compounded by institutional racism (Holroyd, 2015). Autistic individuals are more likely to experience (poly)victimization, including being four times more likely to experience physical and psychological abuse from adults as children, 27 times more likely to experience teasing, and seven times more likely to experience sexual victimization (Weiss and Fardella, 2018). At the extreme end of the victimization—autistic children are more likely to die to filicide (Lucardie, 2005). Autistic lives are marked by an often-astounding excess stress burden across the life span.

Considering the study by Hirvikoski et al. (2016), I chose to study mental health and minority stress because people like me were (and still are) dying to suicide in their droves. To be clear, wanting a better future for my community is a value, and my work embodied it from the very beginning. I was propelled by values. How can you belong to a community who is actively suffering, and not want to make it better anyway that you can?

At this point, I discovered both the vastness of autism literature, and the endlessness of its dehumanization of autistic people. Dehumanization is defined as the denial of full humanness to others (Haslam, 2006), the denial of a group's community or identity (Kelman, 1973), exclusion of a group from moral boundaries (Opotow, 1990), the denial of a group's ability to experience complex emotions (Leyens et al., 2000), or the denial of specific traits which are said to unite all humans, or separate non-human animals from humans (Haslam, 2006). These traits include civility, refinement, moral sensibility, rationality or logic, maturity, responsiveness, emotional warmth, individuality, depth, or agency (Haslam, 2006). Dehumanization and exclusion from moral boundaries serve to facilitate the permissibility of violence against a group (Opotow, 1990; Haslam and Loughnan, 2014), something which is reflected in how freely, and without restraint the literature debates the eugenic removal of autistic people. In talking about violence, I include physical, psychological, emotional, and verbal violence, including interpersonal victimization (Griffiths et al., 2019), and also systemic violence perpetrated through societal systems such as research (Teo, 2010). Dehumanizing and/or stigmatizing research or narratives are both an act of violence against autistic people, but also facilitate the permissibility of more intimate violence such as interpersonal victimization. As I have pointed to in both empirical (Botha et al., 2020) and theoretical papers (Botha, accepted)—dehumanization of autistic people in research is endemic. Below I highlight some key quotes, and rather than summarize them I include them so that there is no ambiguity or debate about “interpretation” in how research discusses autism and/or autistic people. The quotes below highlight this sort of research-based violence with their dehumanization of autistic people, and are all specific examples of quotes I was exposed to during my MSc and Ph.D.:

“*Originality is attractive even in the domestic sphere as long as it does not topple over into uncomfortable eccentricity. However, it is only a few people with ASD [autism spectrum disorder] who combine originality with high levels of intelligence and industry who are likely to make a sufficiently sustainable, salient contribution that their absence might be considered unaffordable*” (Tantam, 2009, p. 219).

“*…autism spectrum disorders (ASD) have become preferred labels for problems reaching the criteria for disability for a variety of reasons, including trends in what is considered chic and the increasingly common abandonment of prevention as a goal… We are also concerned that positive views of disability [*including ASD*] inadvertently undermine prevention… preventing them likely becomes a matter of little concern. If being born with a disability is not also seen as being undesirable*—*in fact, as a birth defect*—*then we fear there will be little reason to prevent such anomalies. If we care about the quality of life of people with disabilities and their loved ones we will certainly do all we can to… prevent others from having a disability”* (Kauffman and Badar, 2018, p. 53).

“*In general, it seems that neither apes nor children with autism have*—*at least not to the same extent as typically developing human children*—*the motivation or capacity to share things psychologically with others. This means that they both have very limited skills for creating things culturally with other persons”* (Tomasello et al., 2005, p. 687).

“*It is our intention to show that people with ASD exhibit less marked domesticated traits at the morphological, physiological, and behavioral levels… specifically, in adults the abnormal shape of the ears is robustly associated with autistic traits, with higher scores correlating with poorer functioning (Manouilenko et al., 2014). Regarding the changes in the orofacial region, prepubertal boys with ASD show significant differences in facial morphology compared to typically developing (TD) boys (Aldridge et al., 2011)… This distinctive facial phenotype is more pronounced in subjects with severe symptoms, significant cognitive impairment, and language regression (Obafemi-Ajayi et al., 2015). Concerning tooth peculiarities, children with ASD show greater abnormalities in dentition, including missing teeth, diastemas, or reverse overjets (Luppanapornlarp et al., 2010) … Regarding the behavioral traits associated with the domestication syndrome, we wish to highlight that aggressive behaviors are frequent in children with ASD (with about 25% of them having scores in the clinical range), and correlate with lower cognitive outcomes (Hill et al., 2014). Children with ASD display more reactive than proactive aggression attitudes (Farmer et al., 2015).”* (Benítez-Burraco et al., 2016, p. 1).

“*The person with autism's difficulty is more profound, making the possibility of identifying with a community more daunting. While it is true that communities of persons exist, disabled or otherwise, it is not the case that a community of autistic people is one of them. There is not, nor could there be a community of autistic people, since a failure of ‘theory of mind’ would preclude being a part of any community”* (Barnbaum, 2008, p. 157).

“*One way to describe the social impairment in Asperger syndrome is as an extreme form of egocentrism with the resulting lack of consideration for others… This egocentrism seems to present a huge difficulty in forming successful long-term interpersonal relationships. Spouses and family members can experience bitter frustration and distress. They are baffled by the fact that there is no mutual sharing of feelings, even when the Asperger individual in question is highly articulate.”* (Frith, 2004, p. 676).

“*Autistic children are severely disturbed. People seem to be no more than objects to them… You see, you start pretty much from scratch when you work with an autistic child. You have a person in the physical sense—they have hair, a nose and a mouth—but they are not people in the psychological sense*.” (Lovaas, 1974).

“*Autistic integrity seems more akin to the type of integrity informing environmentalists' familiar demands for consumer and communal responsibility toward non-human animals”* (Russell, 2012, pp. 169–170).

“*We have argued above that if the mechanism which underlies the computation of mental states is dysfunctional, then self-knowledge is likely to be impaired just as is the knowledge of other minds. The logical extension of the ToM deficit account of autism is that individuals with autism may know as little about their own minds as about the minds of other people… Autism is a devastating disorder because it disrupts not only understanding of others and their social relationships, but also understanding of self* .” (Frith and Happe, 1999, p. 7 and 19).

I became not just a triad of impairments, or someone who lacked the ability to infer the minds of others, or empathize, but something that was described in terms of categorical sub-humanness—incapable of culture, friendship, community, and reciprocity; someone who is less domesticated, more aggressive, an economic burden, with integrity equivalent to non-human animals alone. I balanced sitting exams, with sifting through “objective” accounts of my complete insufficiency as a human-being, often getting lost in the most egregious descriptions of what it “means” to be autistic. But it was okay, because some of these very same articles employed person-first language—the language I was repeatedly told made people like me *more* human. I wondered, as I have for years, if that is even something you can forget when you look at autistic people. The literature taught me that certainly it is something some can “forget” while writing about autistic people, and that person-first language is the placation autism researchers offer themselves in the same breath as refusing to acknowledge that there is any human in autistic people at all.

When analyzing the results from my MSc study I found that exposure to minority stress *does* predict significantly worse well-being and higher psychological distress in the autistic community (Botha and Frost, 2020), including exposure to victimization and discrimination, everyday discrimination, expectation of rejection, expectation of rejection, outness (disclosure), concealment (masking of autism), internalized stigma, and it explains a large and significant proportion of the variance—in lay-man's terms—the constant marginalization of autistic people is contributing to high rates of poor mental health. Aside from this, I noticed that despite being normally distributed (and not containing outliers), the mean psychological distress score was above the cut-off for indicating severe psychological distress (Kessler et al., 2003). Between the sadness of these findings and being exposed to all of these disturbing accounts of autism I considered (albeit briefly), giving up on academia all together without pursuing my Ph.D.

At my first conference, in the first year of my Ph.D. I enthusiastically explained my research while standing next to my first real research poster. The poster detailed my MSc paper which found that a large proportion of the variance of poor mental health and well-being could be explained by exposure to minority stress, and parts of my first study of my Ph.D.—a qualitative investigation into autistic community connectedness. A conference delegate asked, “why did you do this research?.” I disclosed being autistic, and pointed to the clear need for the research and the delegate's response was “oh… are your supervisors? I just worry that you might be biased in, like… you know… this research?.” In that moment I recalled reading all the accounts that I detailed above—all these “objective” accounts of my sub-humanness. I asked the delegate what they meant, and they explained further that they are not necessarily sure that an autistic person would be best placed to talk about autism, but that it should be fine as long as I have non-autistic research supervisors checking over my work, to make sure that I am being “fair,” and “equal” in my representation of autistic people. I was discounted again.

During my first year of my Ph.D. I submitted my first paper to be published, on minority stress and mental health in the autistic community—it was desk rejected by the first journal I sent it to, because there are (apparently) not enough autistic people in general for it to be important to a general audience, making it out of the scope of the journal—a journal which regularly publishes arguably niche research about other minority groups. When it goes out for review elsewhere the editor returns the review comments with a long paragraph about why I have objectified autistic people by using identity first language, and that I really should not define autistic people by their autism alone, and that if I said “person with autism” I would be re-iterating autistic people's personness. One reviewer asked me to double check the psychological distress scores because the scores being normally distributed would indicate a very distressed sample. I double-checked the data—it is still accurate, normally distributed, and yes, autistic people are still not okay.

It was toward the middle of my second year on my Ph.D. that I entered a crisis of faith in Psychology because it seemed like Psychology was built as a pyramid of playing-cards—ready to collapse at any moment. The predominant default in my MSc education was a steadfast positivistic Psychology. I was taught about statistics, experimental design, the replication crisis, and the movement toward “objective” measures like fMRI, and neuroscience. I was taught about statistical reliability and validity, but rarely about meta-theory, and what underpins the whole field of Psychology. Indeed, this uncomplicated picture of Psychology was one underpinned by positivism (and its successor logical empiricism) which aims for deduction through controlled experimentation using operationalized variables, and aims for reproducibility, objectivity, and value-freedom (Tolman, 1992). The application of the scientific process is said to create value-free objective knowledge (Fondacaro and Weinberg, 2002) whereby their evaluation transcends social and cultural predilections and represents an aptly named “view from nowhere” (Nagel, 1989). To juxtapose this extensive education on positivistic Psychology was a limited exposure to qualitative Psychology—predominantly through an interpretivist lens—and some discussion of meta-theory more deeply during one module on the conceptual and historical issues of Psychology alone.

Despite this, as multiple authors have highlighted, and as I realized, there is a lack of transparency in quantitative methods, partly because of the assumption of objectivity awarded almost without question, to statistical work (McGuire, 1983; Gigerenzer, 2004; Tebes, 2005; Bayarri et al., 2016). Yet, data is manipulated without disclosure for many reasons (Gigerenzer, 2004; Cumming, 2014). This manipulation goes beyond carelessness, given that in a study of 697 articles, researchers found that while 63% had inaccurate *p*-values, 20% of which were so grossly misrepresented that it would have changed the decision about significance in favor of authors hypotheses (Veldkamp et al., 2014). This is before acknowledging the fact that interpretation is an action and acknowledging that data do not speak for itself (Teo, 2010), meaning that *even* if you have applied the scientific method you cannot take the scientist out of the science. We discuss data as if it “speaks” for itself, rather than as the product of our measurement, design, and creation, all of which are predicated on the assumptions brought into the investigation (Barad, 2007). As such, all of science is entangled with the people who create it (Barad, 2007). Despite any claims to value-neutrality, and science status Psychology has a bigotry that I highlight elsewhere (Botha, accepted), such as racism (Schaffer, 2007), ableism (Scully and Shakespeare, 2019), and homophobia (Mohr, 2009) all of which involves centring psychology in social and cultural values, without acknowledgment. In particular, for example, Black people have long been racialized by psychologists, with a determined effort to establish group inferiority based on skin colour in empirical psychology (Teo, 2011), while sexuality and gender minorities have been pathologized, misgendered, and devalued (Bayer, 1987; Ansara and Hegarty, 2012). I became disillusioned during my second year specifically because all of these processes (research design, statistical analysis, and quantitative psychology) were sold somewhat as the “objective” saviors of an otherwise previously “subjective,” anti-scientific field. Yet, these processes also formed part of the process of autistic marginalization—these theories and studies have themselves have been based upon empirical findings.

There were hundreds of discussions about the replication crisis, and none about the implicit power of claiming that psychology is value-free, nor the violence that it is inflicted on marginalized groups. A violence I saw and experienced first-hand as an autistic doing autism science. Despite my thesis being an empirical thesis, I spent years reading both broadly and deeply on philosophy of science to reconcile my discomfort with Psychology, and my discomfort of being an autistic person creating autism science. Some colleagues told me that they cannot understand why I am so hung up on this. I am told that I am over-thinking this. But I was determined to reconcile this because Psychology has been a field that has shown an abhorrent lack of respect for people like me. I have felt like a traitor to contribute to the field who not only made me into a category, but who also categorically dehumanized me. Drawing on the idea of Ian Hacking once more: autism is not a natural category—it is a category created in the shadow and context of social and cultural values, and one which only came into the public consciousness because of Psychology and related fields (Hacking, 2006). There is no objectivity in this process—only a position from which we look at certain people. I focused so deeply during my Ph.D. on what constitutes objectivity, because on one hand I have been repeatedly told that I cannot be it, while on the other people using value-laden language have been upheld uncritically as being the paragon of objectivity. I surfaced from this crisis abandoning any claim to objectivity in the opening paragraphs of my thesis in favor of radical transparency (Botha, 2020)—acknowledging what I was doing, why I was doing it, and how I was doing it.

During my last year, I submitted another paper (qualitative) first as a pre-print and then for review. It has a section on the dehumanization of autistic people in research—a section that I highlight with specific examples dating over 60 years. Three things happened. Firstly, I received an irate email from an author declaring me slanderous for characterizing their work as dehumanizing, saying that I should remove the reference to the work if I cannot understand it. Secondly, when peer reviews returned, a reviewer asked that I say “not all autism research is dehumanizing” as if any amount would be okay. Thirdly, the *pre-print* is peer-reviewed post-publication of the paper on a reviewing site—in the pre-print I do not disclose being autistic, but in the final publication I do—the public review states the following:

“*There is a potential bias due to the lead researcher completing the interviews and having autism themselves. This should be stated in the article.”*

At this point, I am no longer an undergraduate, I have been awarded my BA, MSc, and Ph.D., I have three peer-reviewed publications with a fourth and fifth on the way—I am still being told that I do not understand the literature, and that I am biased. At a certain point it becomes easy to see that it was never about my education or engagement with the literature, it is about my autism—we do not trust, nor want autistic people to talk about autism. First-hand accounts go ignored, and when they defy the expectations of the experts the writers are dismissed as potentially *not even* autistic (Frith, 2004). Our narratives are described as unreliable *because* of our autism (Frith and Happe, 1999). If we do not have qualifications in the field we are not qualified to speak to our *own* existence, and yet, even when we are we are biased anyway. Epistemic injustice pervades autism research in a way that only ever marginalizes autistic people in knowledge creation while providing an almost all-encompassing blanket of protection for non-autistic researchers—non-autistic people have an assumed objectivity that means they do not have to defend their involvement in the creation of knowledge.

## Values, Transparency, and Rigor

I have come to believe that all research is driven by values, and instead it is not the presence of values which biases research, but instead the transparency of said values. Values sustain my need for accepting autism, and values sustain the researchers who believe that eradicating autism is a necessity or public good. It is about being honest about which values we are embedding in our work and forgoing hiding behind a guise of objectivity. As such, I aim for rigor. Rigor here, is defined as ethical, robust, and thorough research design which addresses research questions in a transparent and repeatable way. This idea of rigorous applies equally between quantitative and qualitative psychology depending on the aim of each individual study. For quantitative research this can mean having methods that reduce the potential for research-design based bias such as random allocation, double-blinded study design, hypothesis registration, and data-sharing. For required qualitative research, this can involve having methods that ensure accessibility of design such multiple ways of partaking beyond speaking, a robust design and coding procedure which does not favor the narrative of specific participants, and of course transparency. Across qualitative, quantitative, empirical, and theoretical work, it means prioritizing transparency and reflexivity. As such, instead I work to lay claim to rigor over objectivity, because I do not believe that any research has the ability to be objective in the sense of value-free.

## “I'm Not Talking About you” and “Not all Autism Research…”: Anger

As an autistic person, when I talk to people about the dehumanization of autistic people in research, researchers are quick enough to exclaim “but I am not talking about you!.” Indeed, I have often been the exception to the rule. But autistic people always are—one moment researchers will engage with autistic people, and we will be afforded a temporary personhood, that extends only to the life span of the conversation. We are not taken as evidence of the fallibility of the field, we are, all of us, outliers in the metaphorical sense, and the metaphorical sense alone. I say metaphorical because by the time you have so many exceptions to the rule, statistically, it stops being an outlier. There has been a tradition since the birth of autism to be selective about which autistic receives rights and recognition, with Hans Asperger himself relegating some to death (Czech, 2018), and even now, we *still* eagerly discuss which autistics we can afford (Ganz, 2006; Tantam, 2009)—but it is *never me*. It is always the “other kind” of autistics. Researchers always like to say that they are talking about the *hard* autism, and not me, as if they are privy to all the iterations of my autism from babyhood to adulthood. Everyone is quick to fill in my past based on my present and they usually miss the mark—the very same way that when I was younger and struggled, I was told I was going nowhere. They are two sides of the same coin.

With regards to dehumanization of autistic people in research—I am not the first, nor will I be the last autistic who struggles with how dehumanizing, objectifying, or alienating autism research is (Luterman, 2019; Rose, 2020; Michael, 2021). To be involved in autism research when you are autistic, is to constantly experience the aggression of a field which has yet to come to terms with its own ableism. It is not only to face an ableist academia, but one that fails to acknowledge that there is even a problem. Some academics (both autistic and not) have written about the dehumanizing nature of the autism academy (Gernsbacher, 2007; Cowen, 2009; Milton, 2016) but more widely, there is an astounding lack of awareness that we are speaking or writing about, and constituting people—words, descriptions, and constructions of people will have wider consequences. I was in no way surprised when mid-Ph.D. a study was published showing that autistic people are dehumanized by the general population (Cage et al., 2018).

I *feel* angry and frustrated at these objectifying dehumanizing narratives and have since I was an undergraduate. But I am not meant to say this. I have been told many times to leave my emotions at the door. It is not “professional” to engage emotively with science. My sadness is taken as evidence of bias. I am told to be objective, and separate myself from the descriptions, the violence, and dehumanization. Instead, I have leaned-in—in a radical act of defiance I am transparent, vulnerable, and honest. I refuse to experience this anger alone, or in silence anymore because it functions to uphold the status quo. Reflexivity is meant to unsettle the status quo (Pillow, 2003), and I use my own vulnerability and openness to unsettle it further by refusing to remain quiet, compliant, or passive while my community experiences the willing oppression of violent research. I lean into my emotions because they inform my values, keep me tied to the autistic community, generate my sense of epistemic responsibility to the community I come from. I am open because when autistic students (whether undergraduate or postgraduate) approach me to ask how I handle the experience of feeling and living these accounts, they express a loneliness that silence only serves. I now have a policy of honesty and I tell them: I feel angry.

## Entanglement

The idea of science being entangled with measurement is not radical—it is a Bohrian understanding of science where we acknowledge that the act of measuring a phenomenon can change it (Barad, 2007). Autism has never been free from the people who created it, or who continue to create it. The people who delineated us from any other constitution, or patterns of behaviors by grouping us together based on our behavior and communication, have a routine history of perpetuating the stereotypes that limit us, degrade us, and form the basis of some degree of our oppression. This includes denying us any epistemic authority to give meaning to what it *means* to be autistic (Frith and Happe, 1999; Frith, 2004) so as to remove access to challenging the constant barrage of deficit and disease framings. Another autistic academic said it best: “autism discourse and I are co-constituted” (de Hooge, 2019). As an autistic I feel the reverberations of the scientific discourse into my personal life—it radiates into social media, informs stereotypes, creates discourses, and ideas of autism that comes to grow amongst our families, friends, colleagues, community, and the strangers we encounter.

As a critical realist (expanded on here Botha, in review), I do not conflate these ideas of autism with what autism *actually* is—autism itself is *not* created by discourse. Rather, these ideas of autism will have materials consequences for autistic people as they become barriers and challenges. Autistic people feel trapped by the stereotypes society has of autism (Treweek et al., 2018), but a lot of these originate in research and trickle down into the press—including the idea that we lack empathy or theory of mind (Gernsbacher, 2017). We *are* a part of the discourse, in that we are created in people's minds by it, and affected by it in our everyday lives—and yet some are quick to point out that *some* autism research is not for autistic people or their families, but rather *about* autistic people, and for academics (as if mutually exclusive) (Baron-Cohen, 2009a). Regardless of whether autistic read these accounts (and both autistic people and autistic autism researchers can and do), there are consequences that the rest of us will come to experience anyway, as it cascades into the media and our lives. Ableism is entangled with our measurements of autism—we create deficit focused measures, which only could measure deficits and use it to confirm ideas of that autism *is* deficit and from it we create deficits narratives that pervade almost all conversations of autism. Autistic people are inherently entangled with these discourses.

As another openly autistic academic put best: “These shitty narratives persist… because their rhetorical power derives from the figure of the autistic as unknowable, as utterly abject and isolated and tragic, as a figure whose actions are construed less like actions and more like neuronally willed middle fingers” (Yergeau, 2018, p. 3). The idea of autistic people as lacking in intentionality, theory of mind, and empathy has left us as objectified at best, dehumanized at worst, and has yet to make for reliable science too. The theory that autistic people have some sort of impaired theory of mind is and has been constantly plagued by innumerable empirical failings (Gernsbacher and Yergeau, 2019) and yet forms the basis for many early interventions aimed at making us “people,” or at least people enough to be classed as having been remediated by medicine. But, poor theory has made for poor evidence, with interventions based on theory of mind showing little efficacy anyway (Fletcher-Watson et al., 2014), while other early intervention research too shows little efficacy (Sandbank et al., 2020), and an astounding rate of conflicts of interest (Bottema-Beutel et al., 2020). But, however inaccurate, flawed, or (increasingly) useless these theories are for explaining autism, it seems we cling to them because we cannot get past an idea of autistic people as blank pages, empty shells, bare slates, who cannot think about themselves, nor other people, who are less capable with empathy, socialization, who are wrapped up in restrictive, repetitive behaviors—this is autism academia's great legacy.

## Emerging

An ethical and reflexive approach to creating and discussing autism science is sorely missing—and the lack of it has changed the course of my experience through my undergraduate, into my masters, and throughout my Ph.D., and now beyond. Like many other autistic academics, I did not have the privilege of *just doing* science. I rote-learned my own dehumanization to get my undergraduate, exposed myself to the most damaging literature to get an MSc, and experienced my own systematic dehumanization in the process of getting my Ph.D. For many years, I struggled to make sense of the seeming fragility of Psychology, the marginalizing constructions of objectivity, and the violence perpetrated by a positivistic Psychology (explored in detail here (Botha, accepted). When I say emerging here, I make use of critical realism, and how phenomena emerge from many layers of reality—from the “real,” to the social, and cultural. I have emerged from my Ph.D. to understand something I did not previously—Psychology is not precarious, or a house of cards. Psychology is robust, and in some ways unchanging, because it was *designed* to function in this way. Psychology, especially constructed as a science was designed to objectify, which is why it has been so *thorough* at perpetuating racism, transphobia, ableism, homophobia, and bigotry. It was designed to center non-marginalized peoples' perspectives of the marginalized—and it was designed to leave no room for recourse. This is why non-autistic researchers can so readily engage arguments of objectivity to silence the meaning from autistic autobiographies, and autistic researchers (Frith, 2004; Hacking, 2009)—it maintains the status quo.

Yet, I emerge—a product of autism, discourse, activism, and academia, creating pockets of agency, to resist. Much like those who challenge the status quo to produce critical autism literature (Woods and Waldock, 2020). I follow in the footsteps of openly autistic academics, whose visibility was the only reason I saw this as a viable career—such as Damian Milton, or Steven Kapp. More than ever, I hope to hold the door open for other autistic people to follow in our footsteps, and to reclaim knowledge production. For this to be truly sustainable however, we need to speak openly about the hospitability of the field, and as it stands, it is barely hospitable, if at all. We have emerged, but we carry burdens that non-autistic autism researchers do not face. This needs to be acknowledged for any sort of sustainable contribution of autistic people to be realized—I worry that we throw ourselves in, we burnout, and are disposed.

Those with the most power in the field ought to share this burden by challenging the system that creates autistic dehumanization, by challenging the language, the systemic marginalization, by listening to autistic people in research when they say, “this is not okay,” and more than ever, by talking about “objectivity.” This requires engaging in a constant dynamic learning process as society, culture, and our ideas of autism change, to ensure that we do not become static markers of this time or place. Even the most progressive ideas we have of autism now might be regarded as regressive in a few years from now, and lest we forget to grow and adapt we will perpetuate a similar violence. As such, we have a responsibility to make our own self-change and learning happen alongside our reflection, and a duty to try to ensure our colleagues do the same (including highlighting when someone perpetuates violence within the field). Challenging the system means challenging the permissibility of perpetuating poor, outdated, or harmful science, including as what is defined as such changes over time. As such, it is our responsibility to learn, grow, and to hold colleagues accountable for the same, such that in no time or place again, it is okay to dehumanize or victimize autistic people.

While some days I have hope that there is change—from an increased focus on participatory research, to what seems to be an increased presence of autistic people both in and leading autism research, as well as what appears to be change from long-standing autism academics who are slowly abandoning person-first language, the puzzle piece, and dehumanizing (and inaccurate) theories of autism—the days that I feel hope from this are few and far between. This is particularly because as I have become more prominent in my role, and increasingly work with students, research assistants, or receive communications from autistic people all over the world, I notice still, how many autistic people drop away from autism research—and most reference just how harmful they have found the field. When I encounter people in this situation, I always do my best to make clear that the system is broken, and no one should have to withstand it. I reiterate that it is the fields loss (and it always is), and that it takes tremendous strength to know one's own best interests and to walk away from a field to which they have often already dedicated years of their life. I am also honest and tell them that I honestly think about leaving academia completely myself too (often), despite what—from the outside—looks like an otherwise great career trajectory. So, more often than not, I do not feel hope for the field despite this progress because I see all the empty space where incredible autistic researchers have left, and I feel impatient for change to come more quickly because I am so desperate for these gaps to happen less often.

## Reflexivity

This article is the product of multiple years of ongoing ruminating reflexivity. Elsewhere I discuss the sheer importance of reflexive practice *theoretically* for theoretical and empirical (whether quantitative or qualitative) work (Botha, accepted), especially as a way of instigating change. Instead, here I make a call to action—all researchers, please, engage with your own values, interrogate them, unpick them, doubt yourself, acknowledge your fallibility, acknowledge your mistakes, apologize, and engage with autism reflexively. There is no greater responsibility than constituting people—and we as psychologists do this (Hacking, 2006).

There have been times in which I have been compelled to do things in a certain way because that is how the field or Psychology “works.” There have been movements where my insider knowledge of the autistic community has come second to the methodolatry of Psychology—the retainment of an idea of method validity has been prioritized over the effect of such methods on my community. I have been urged to only include diagnosed autistic people to make it “more valid” easier to publish (despite the widely acknowledged racial, economic, class, and gender disparities in diagnosis) (Mandell et al., 2009; Shefcyk, 2015; Newschaffer, 2017). I have been pushed toward deficit-based definitions, concepts, and language—and have a lot of regret for when I did not push back. I have made my own mistakes—including using functioning labels in my very first article (Botha and Frost, 2020) because it was “the ‘done’ thing.” My responsibility after this was to learn, push back harder the next time, and apologize unreservedly for the damage such language has the potential to cause—and as such, I am so completely sorry. My entire thesis did not meet the standards I have now for research despite the elements of agency I tried to embed throughout; to say this is not to devalue my work, but rather, it is to acknowledge learning and growth. If I could do my Ph.D. over, I would make it a participatory project, and embedded autistic voices beyond my own more throughout all of the work. I worked with the tools that I had at the time, but it does not excuse where I went wrong.

In the end, my thesis (Botha, 2020) showed that autistic community connectedness buffered against some of the effects of minority stress and was related to better mental health over time. Yet, I worry constantly that by trying to measure a function of autistic community connectedness, that I objectified it, in a way not dissimilar to the way people objectify autistic people—especially if others come to conflate the *function* of autistic community connectedness with its *value*. I studied autistic community connectedness, because I was worried that to only study minority stress would be to see only the worst of what happened to autistic people, and not appreciate our lives as a whole—which are much bigger than our trauma. But, to me, the numbers only explain a mechanism—the real joy, the real value, and the beauty of the autistic community was captured in my very first study. Autistic people talked about the autistic community with such a warmth, brightness, and with hope. The vibrant stories of belongingness, friendships, and political strength tell you exactly what you need to know about the value of such a community. This is something, that its function cannot, and should not even tell you.

## Conclusion

In my title, I ask “academic, activist, or advocate?”—and my answer is that I am all three. You cannot belong to a community that suffers from violence, marginalization, and suicide and not be. In my introduction I tell readers all the different types of autistic people I have been in the eyes of the clinicians and professionals who deemed my future limited or limitless because whenever an autistic person tells you anything about what it *means* to be autistic that is not just a list of impairments or limitations, we are told that we must have the “easy” autism. I laid this out so transparently to challenge the idea that just because we (autistic people) have fought to be included in autism research does not mean that you can picture where we have been (including how we experienced our own autism growing up). To conclude: I will not leave my values at the door of the academy—I refuse. I refuse to abandon my community and to engage in the complicit silence. Instead, I offer up transparency, openness, a constantly reflection, and learning. Instead, I make space for growth, action, and strive toward a social change for autistic people. It seems there is nothing more radical than that.

## Author Contributions

The author confirms being the sole contributor of this work and has approved it for publication.

## Funding

This work was supported by the University of Surrey, Department of Psychology, and its dissemination by the Economic and Social Research Council (grant number ES/V012347/1). Lastly the open access publications fees are provided by the University of Stirling APC fund.

## Conflict of Interest

The author declares that the research was conducted in the absence of any commercial or financial relationships that could be construed as a potential conflict of interest.

## Publisher's Note

All claims expressed in this article are solely those of the authors and do not necessarily represent those of their affiliated organizations, or those of the publisher, the editors and the reviewers. Any product that may be evaluated in this article, or claim that may be made by its manufacturer, is not guaranteed or endorsed by the publisher.

## References

[B1] Ansara Y. G. Hegarty P. (2012). Cisgenderism in psychology: pathologising and misgendering children from 1999 to 2008. Psychol. Sex. 3, 137–160. 10.1080/19419899.2011.576696

[B2] Baldwin S. Costley D. Warren A. (2014). Employment activities and experiences of adults with high-functioning autism and Asperger's disorder. J. Autism Dev. Disord. 44, 2440–2449. 10.1007/s10803-014-2112-z 24715257

[B3] Barad K. M. (2007). Meeting the Universe Halfway: *Quantum Physics and the Entanglement of Matter and Meaning*. Duke University Press.

[B4] Barnbaum D. R. (2008). The Ethics of Autism: Among Them, But Not of Them. Bloomington, IN: Indiana University Press.

[B5] Baron-Cohen S. (2009a). Does autism need a cure? Lancet 373, 1595–1596. 10.1016/S0140-6736(09)60891-6

[B6] Baron-Cohen S. (2009b). Autism: the empathizing-systemizing (E-S) theory. Ann. N. Y. Acad. Sci. 1156, 68–80. 10.1111/j.1749-6632.2009.04467.x 19338503

[B7] Baron-Cohen S. Bolton P. (1993). Autism: The Facts. Oxford; New York, NY: Oxford University Press.

[B8] Baron-Cohen S. Leslie A. M. Frith U. (1985). Does the autistic child have a “theory of mind” ? Cognition 21, 37–46. 10.1016/0010-0277(85)90022-8 2934210

[B9] Bayarri M. J. Benjamin D. J. Berger J. O. Sellke T. M. (2016). Rejection odds and rejection ratios: A proposal for statistical practice in testing hypotheses. J. Math. Psychol. 72, 90–103. 10.1016/j.jmp.2015.12.007 30713353 PMC6358203

[B10] Bayer R. (1987). Homosexuality and American Psychiatry: The Politics of Diagnosis. Princeton, NJ: Princeton University Press.

[B11] Benítez-Burraco A. Lattanzi W. Murphy E. (2016). Language impairments in ASD resulting from a failed domestication of the human brain. Front. Neurosci. 10, 373. 10.3389/fnins.2016.00373 27621700 PMC5002430

[B12] Botha M. (2020). Autistic Community Connectedness as a Buffer Against the Effects of Minority Stress. Ph.D. Thesis, University of Surrey. 10.15126/THESIS.00854098

[B13] Botha M. (accepted). Critical realism, community psychology, and the curious case of autism: a philosophy and practice of science with social justice in mind. J. Community Psychol. 10.1002/jcop.22764 34897720

[B14] Botha M. (in review). Critical realism, community psychology, and the curious case of autism: a philosophy and practice of science with social justice in mind. J. Community Psychol. 10.1002/jcop.22764 34897720

[B15] Botha M. (in review). “Community psychology as reparations for violence in the construction of autism knowledge,” in The Critical Autism Handbook, 1st Edn. eds D. Milton S. Ryan (Routledge).

[B16] Botha M. Dibb B. Frost D. (2020). “Autism is me”: An investigation of how autistic individuals make sense of autism and stigma. Disabil. Soc. 10.1080/09687599.2020.1822782. [Epub ahead of print].

[B17] Botha M. Frost D. M. (2020). Extending the minority stress model to understand mental health problems experienced by the autistic population. Soc. Ment. Health 10, 20–34. 10.1177/2156869318804297

[B18] Bottema-Beutel K. Crowley S. Sandbank M. Woynaroski T. G. (2020). Research Review: Conflicts of Interest (COIs) in autism early intervention research – a meta-analysis of COI influences on intervention effects. J. Child Psychol. Psychiatry 62, 5–15. 10.1111/jcpp.13249 32353179 PMC7606324

[B19] Cage E. Di Monaco J. Newell V. (2018). Understanding, attitudes and dehumanisation towards autistic people. Autism 23, 1373–1383. 10.1177/1362361318811290 30463431

[B20] Chapman R. (2020). The reality of autism: On the metaphysics of disorder and diversity. Philos. Psychol. 1–21. 10.1080/09515089.2020.1751103

[B21] Cowen T. (2009). Autism as Academic Paradigm. The Chronicles of Higher Education. Available online at: http://www.chronicle.com/article/Autism-as-Academic-Paradigm/47033 (accessed September 07, 2021).

[B22] Cumming G. (2014). The new statistics: why and how. Psychol. Sci. 25, 7–29. 10.1177/0956797613504966 24220629

[B23] Czech H. (2018). Hans Asperger, national socialism, and “race hygiene” in Nazi-era Vienna. Mol. Autism 9, 1–43. 10.1186/s13229-018-0208-6 29713442 PMC5907291

[B24] de Hooge A. (2019). Binary boys: autism, aspie supremacy and post/humanist normativity. Disabil. Stud. Quar. 39:2. 10.18061/dsq.v39i1.6461

[B25] De Rubeis S. He X. Goldberg A. P. Poultney C. S. Samocha K. Ercument Cicek A.. (2014). Synaptic, transcriptional and chromatin genes disrupted in autism. Nature 515, 209–215. 10.1038/nature13772 25363760 PMC4402723

[B26] Evans (2011). The Life We Choose: Shaping Autism Services in Wales. Available online at: file:///C:/Users/mb00786/Downloads/TheLifeWeChoose_Eng_FLR.pdf

[B27] Evans B. (2013). How autism became autism: the radical transformation of a central concept of child development in Britain. Hist. Human Sci. 26, 3–31. 10.1177/0952695113484320 24014081 PMC3757918

[B28] Fletcher-Watson S. Adams J. Brook K. Charman T. Crane L. Cusack J.. (2019). Making the future together: shaping autism research through meaningful participation. Autism 23, 943–953. 10.1177/1362361318786721 30095277 PMC6512245

[B29] Fletcher-Watson S. McConnell F. Manola E. McConachie H. (2014). Interventions based on the Theory of Mind cognitive model for autism spectrum disorder (ASD). Cochrane Database Syst. Rev. 2014, CD008785. 10.1002/14651858.CD008785.pub2 24652601 PMC6923148

[B30] Fondacaro M. Weinberg D. (2002). Concepts of social justice in community psychology: toward a social ecological epistemology. Am. J. Commun. Psychol. 30, 473–492. 10.1023/A:1015803817117 12125778

[B31] Frith U. (2004). Emanuel Miller lecture: confusions and controversies about Asperger syndrome. J. Child Psychol. Psychiatry 45, 672–686. 10.1111/j.1469-7610.2004.00262.x 15056300

[B32] Frith U. Happe F. (1999). Theory of Mind and self-consciousness: what is it like to be autistic? Mind Lang. 14, 82–89. 10.1111/1468-0017.00100

[B33] Frost D. M. Lehavot K. Meyer I. H. (2015). Minority stress and physical health among sexual minority individuals. J. Behav. Med. 38, 1–8. 10.1007/s10865-013-9523-8 23864353 PMC3895416

[B34] Ganz M. (2006). “The costs of autism,” in Understanding Autism, eds S. Moldin and J. Rubenstein (London: CRC Press), 475–502. 10.1201/9781420004205.ch20

[B35] Gernsbacher M. A. (2007). On not being human. APS Obs. 20, 5–32. PMC4266404 25520547

[B36] Gernsbacher M. A. (2017). Editorial Perspective: the use of person-first language in scholarly writing may accentuate stigma. J. Child Psychol. Psychiatry 58, 859–861. 10.1111/jcpp.12706 28621486 PMC5545113

[B37] Gernsbacher M. A. Yergeau M. (2019). Empirical failures of the claim that autistic people lack a theory of mind. Arch. Scien. Psychol. 7, 102–118. 10.1037/arc0000067 31938672 PMC6959478

[B38] Gigerenzer G. (2004). Mindless statistics. J. Soc. Econ. 33, 587–606. 10.1016/j.socec.2004.09.033

[B39] Glynne-Owen R. (2010). Early intervention and autism: the impact of positivism and the call for change. Int. J. Child. Rights 18, 405–416. 10.1163/157181810X497431

[B40] Griffiths S. Allison C. Kenny R. Holt R. Smith P. Baron-Cohen S. (2019). The Vulnerability Experiences Quotient (VEQ): a study of vulnerability, mental health and life satisfaction in autistic adults. Autism Res. 12, 1516–1528. 10.1002/aur.2162 31274233 PMC6851759

[B41] Hacking I. (2001). The Social Construction of What? Vol. 8. Cambridge, MA: Harvard Univ. Press.

[B42] Hacking I. (2006). Making Up People. Available online at: https://www.lrb.co.uk/v28/n16/ian-hacking/making-up-people (accessed September 07, 2021).

[B43] Hacking I. (2009). Autistic autobiography. Philos. Trans. R. Soc. B Biol. Sci. 364, 1467–1473. 10.1098/rstb.2008.0329 19528032 PMC2677587

[B44] Hacking I. (2015). “Let's not talk about objectivity,” in Objectivity in Science: New Perspectives from Science and Technology Studies, eds F. Padovani, A. Richardson, and J. Y. Tsou (Cham: Springer International Publishing), 19–33. 10.1007/978-3-319-14349-1_2

[B45] Happé F. Ronald A. Plomin R. (2006). Time to give up on a single explanation for autism. Nat. Neurosci. 9, 1218–1220. 10.1038/nn1770 17001340

[B46] Haslam N. (2006). Dehumanization: an integrative review. Pers. Soc. Psychol. Rev. 10, 252–264. 10.1207/s15327957pspr1003_4 16859440

[B47] Haslam N. Loughnan S. (2014). Dehumanization and infrahumanization. Annu. Rev. Psychol. 65, 399–423. 10.1146/annurev-psych-010213-115045 23808915

[B48] Hirvikoski T. Mittendorfer-Rutz E. Boman M. Larsson H. Lichtenstein P. Bölte S. (2016). Premature mortality in autism spectrum disorder. Br. J. Psychiatry 208, 232–238. 10.1192/bjp.bp.114.160192 26541693

[B49] Holroyd J. (2015). Implicit racial bias and the anatomy of institutional racism. Crim. Just./ Matters 101, 30–32. 10.1080/09627251.2015.1080943

[B50] Home Office (2018). Police Use of Force Statistics, England and Wales: April 2017 to March 2018.

[B51] Johansen (2013). Autism The Latest Global Epidemic. Financial Times. Available online at: http://www.newswire.net/newsroom/financial/74620-autism-the-latest-global-epidemic.html (accessed September 07, 2021).

[B52] Kapp S. K. Gillespie-Lynch K. Sherman L. E. Hutman T. (2013). Deficit, difference, or both? Autism and neurodiversity. Dev. Psychol. 49, 59–71. 10.1037/a0028353 22545843

[B53] Kauffman J. M. Badar J. (2018). Extremism and disability chic. Exceptionality 26, 46–61. 10.1080/09362835.2017.1283632

[B54] Kelman H. G. (1973). Violence without moral restraint: reflections on the dehumanization of victims and victimizers. J. Soc. Issues 29, 25–61.

[B55] Kessler R. C. Barker P. R. Colpe L. J. Epstein J. F. Gfroerer J. C. Hiripi E.. (2003). Screening for serious mental illness in the general population. Arch. Gen. Psychiatry 60, 184–189. 10.1001/archpsyc.60.2.184 12578436

[B56] Leahey T. H. (1992). The mythical revolutions of American psychology. Amer. Psychol. 47, 308–318. 10.1037/0003-066X.47.2.308

[B57] Lenroot R. K. Yeung P. K. (2013). Heterogeneity within autism spectrum disorders: what have we learned from neuroimaging studies? Front. Hum. Neurosci. 7:733. 10.3389/fnhum.2013.00733 24198778 PMC3812662

[B58] Leyens J.-P. Paladino P. M. Rodriguez-Torres R. Vaes J. Demoulin S. Rodriguez-Perez A.. (2000). The emotional side of prejudice: the attribution of secondary emotions to ingroups and outgroups. Pers. Soc. Psychol. Rev. 4, 186–197. 10.1207/S15327957PSPR0402_06

[B59] Lovaas I. (1974). After you hit a child, you can't just get up and leave him; you are hooked to that kid. Psychol. Today 76–84.

[B60] Lucardie R. (2005). Intrafamilial homicide of people with developmental disabilities. Dev. Disabil. Bull. 33, 129–153.

[B61] Luterman (2019). What It's Like to be Autistic at an Autism Research Conference. Available online at: https://www.spectrumnews.org/opinion/what-its-like-to-be-autistic-at-an-autism-research-conference/ (accessed September 07, 2021).

[B62] Mandell D. S. Wiggins L. D. Carpenter L. A. Daniels J. DiGuiseppi C. Durkin M. S.. (2009). Racial/ethnic disparities in the identification of children with autism spectrum disorders. Am. J. Public Health 99, 493–498. 10.2105/AJPH.2007.131243 19106426 PMC2661453

[B63] McGuire W. J. (1983). A contextualist theory of knowledge: Its implications for innovation and reform in psychological research. Adv. Exp. Soc. Psychol. 16:47.

[B64] Meyer I. H. (2003). Prejudice, social stress, and mental health in lesbian, gay, and bisexual populations: conceptual issues and research evidence. Psychol. Bull. 129, 674–697. 10.1037/0033-2909.129.5.674 12956539 PMC2072932

[B65] Meyer I. H. Rossano L. Ellis J. M. Bradford J. (2002). A brief telephone interview to identify lesbian and bisexual women in random digit dialing sampling. J. Sex Res. 39, 139–144. 10.1080/00224490209552133 12476246

[B66] Michael C. (2021). Is being othered a co-occurring condition of autism? Autism in Adulthood 2021, aut.2021.0019. 10.1089/aut.2021.0019 PMC8992897 36601468

[B67] Mikami K. Inomata S. Hayakawa N. Ohnishi Y. Enseki Y. Ohya A.. (2009). Frequency and clinical features of pervasive developmental disorder in adolescent suicide attempts. Gen. Hosp. Psychiatry 31, 163–166. 10.1016/j.genhosppsych.2008.12.003 19269537

[B68] Milton D. E. M. (2016). Disposable dispositions: reflections upon the work of Iris Marion Young in relation to the social oppression of autistic people. Disabil. Soc. 31, 1403–1407. 10.1080/09687599.2016.1263468

[B69] Mohr J. (2009). Oppression by scientific method: the use of science to “other” sexual minorities. J. Hate Stud. 7, 21. 10.33972/jhs.57

[B70] Mottron L. (2021). A radical change in our autism research strategy is needed: back to prototypes. Aut. Res. 2021, aur.2494. 10.1002/aur.2494 34077611

[B71] Nagel T. (1989). The View from Nowhere (First issued as an Oxford University Press paperback). New York, NY: Oxford University Press.

[B72] Newschaffer C. J. (2017). Trends in autism spectrum disorders: the interaction of time, group-level socioeconomic status, and individual-level race/ethnicity. Am. J. Public Health 107, 1698–1699. 10.2105/AJPH.2017.304085 29019763 PMC5637692

[B73] Opotow S. (1990). Moral exclusion and injustice: an introduction. J. Soc. Issues 46, 1–20. 10.1111/j.1540-4560.1990.tb00268.x

[B74] Pellicano L. (2014). Chapter 4. A future made together: new directions in the ethics of autism research. J. Res. Special Educ. Needs 14, 200–204. 10.1111/1471-3802.12070_5

[B75] Perry D. Carter-Long L. (2016). The Ruderman White Paper on Media Coverage of Law Enforcement Use of Force and Disability [White Paper]. Available online at: https://rudermanfoundation.org/wp-content/uploads/2017/08/MediaStudy-PoliceDisability_final-final.pdf (accessed September 07, 2021).

[B76] Pillow W. (2003). Confession, catharsis, or cure? Rethinking the uses of reflexivity as methodological power in qualitative research. Int. J. Qual. Stud. Educ. 16, 175–196. 10.1080/0951839032000060635

[B77] Popper K. R. (2008). The Logic of Scientific Discovery (Repr. 2008 (twice)). New York, NY: Routledge.

[B78] Proff I. Williams G. L. Quadt L. Garfinkel S. (2021). Sensory processing in autism across exteroceptive and interoceptive domains. Psychol. Neurosci. 10.1037/pne0000262

[B79] Redman S. (2009). Don't Write Me Off: Make the System Fair for People With Autism. London: Y Gymdeithas Genedlaethol Awtistiaeth.

[B80] Rose K. (2020). Regarding the Use of Dehumanising Rhetoric. Available online at: https://theautisticadvocate.com/2020/02/regarding-the-use-of-dehumanising-rhetoric/ (accessed September 07, 2021).

[B81] Russell B. (2012). Reflections on ‘autistic integrity.’ Bioethics 26, 164–170. 10.1111/j.1467-8519.2010.01827.x 20497166

[B82] Sandbank M. Bottema-Beutel K. Crowley S. Cassidy M. Dunham K. Feldman J. I.. (2020). Project AIM: autism intervention meta-analysis for studies of young children. Psychol. Bull. 146, 1–29. 10.1037/bul0000215 31763860 PMC8783568

[B83] Schaffer G. (2007). “‘Scientific’ racism again?”: reginald gates, the *mankind quarterly* and the question of “race” in science after the second world war. J. Am. Stud. 41, 253–278. 10.1017/S0021875807003477

[B84] Scully J. Shakespeare T. (2019). Report on the Impact of Ableism in Medical and Scientific Practice (A/HRC/43/41; Bioethics and Disability: Report for UN Special Rapporteur on Disability). United Nations of Human Rights. Available online at: https://www.ohchr.org/EN/Issues/Disability/SRDisabilities/Pages/BioethicsDisabilities.aspx

[B85] Shattuck P. T. Narendorf S. C. Cooper B. Sterzing P. R. Wagner M. Taylor J. L. (2012). Postsecondary education and employment among youth with an autism spectrum disorder. Pediatrics 129, 1042–1049. 10.1542/peds.2011-2864 22585766 PMC3362908

[B86] Shefcyk A. (2015). Count us in: addressing gender disparities in autism research. Autism 19, 131–132. 10.1177/1362361314566585 25789398

[B87] Silberman S. (2015). Neurotribes. Crows Nest, NSW: ALLEN and UNWIN.

[B88] Tantam D. (2009). Can the *World Afford Autistic Spectrum Disorder*? Nonverbal Communication, Asperger Syndrome and the Interbrain. London: Jessica Kingsley Publishers.

[B89] Tebes J. K. (2005). Community science, philosophy of science, and the practice of research. Am. J. Community Psychol. 5, 213–230. 10.1007/s10464-005-3399-x 15909796

[B90] Teo T. (2010). What is epistemological violence in the empirical social sciences?: what is epistemological violence in the empirical social sciences? Soc. Personal. Psychol. Compass 4, 295–303. 10.1111/j.1751-9004.2010.00265.x

[B91] Teo T. (2011). Empirical race psychology and the hermeneutics of epistemological violence. Hum. Stud. 34, 237–255. 10.1007/s10746-011-9179-8

[B92] Timpson E. Great Britain Department for Education. (2019). Timpson Review of School Exclusion.

[B93] Toal F. Daly E. M. Page L. Deeley Q. Hallahan B. Bloemen O.. (2010). Clinical and anatomical heterogeneity in autistic spectrum disorder: A structural MRI study. Psychol. Med. 40, 1171–1181. 10.1017/S0033291709991541 19891805

[B94] Tolman C. W. (1992). Positivism in Psychology Historical and Contemporary Problems. New York, NY: Springer. 10.1007/978-1-4612-4402-8

[B95] Tomasello M. Carpenter M. Call J. Behne T. Moll H. (2005). Understanding and sharing intentions: the origins of cultural cognition. Behav. Brain Sci. 28, 675–691; discussion 691–735. 10.1017/S0140525X05000129 16262930

[B96] Treweek C. Wood C. Martin J. Freeth M. (2018). Autistic people's perspectives on stereotypes: an interpretative phenomenological analysis. Autism 23, 759–769. 10.1177/1362361318778286 29848001

[B97] Veldkamp C. L. S. Nuijten M. B. Dominguez-Alvarez L. van Assen M. A. L. M. Wicherts J. M. (2014). Statistical reporting errors and collaboration on statistical analyses in psychological science. PLoS ONE 9:e114876. 10.1371/journal.pone.0114876 25493918 PMC4262438

[B98] Weiss J. A. Fardella M. A. (2018). Victimization and perpetration experiences of adults with autism. Front. Psychiatry 9, 203. 10.3389/fpsyt.2018.00203 29887806 PMC5980973

[B99] Wing L. Potter D. (2002). The epidemiology of autistic spectrum disorders: is the prevalence rising? Ment. Retard. Dev. Disabil. Res. Rev. 8, 151–161. 10.1002/mrdd.10029 12216059

[B100] Wood C. Freeth M. (2016). Students' stereotypes of autism. J. Educ. Issues 2, 131. 10.5296/jei.v2i2.9975

[B101] Woods R. Waldock K. E. (2020). “Critical autism studies,” in Encyclopedia of Autism Spectrum Disorders, ed F. R. Volkmar (New York, NY: Springer), 1–9. 10.1007/978-1-4614-6435-8_102297-1

[B102] Yergeau M. (2018). Authoring Autism: On Rhetoric and Neurological Queerness. Durham, NC: Duke University Press.

